# A framework for implementing antibiotic stewardship in ambulatory care: Lessons learned from the Agency for Healthcare Research and Quality Safety Program for Improving Antibiotic Use

**DOI:** 10.1017/ash.2022.258

**Published:** 2022-07-04

**Authors:** Sara C. Keller, Sara E. Cosgrove, Melissa A. Miller, Pranita Tamma

**Affiliations:** 1 Division of Infectious Diseases, Department of Medicine, Johns Hopkins University School of Medicine, Baltimore, Maryland; 2 Center for Quality Improvement and Patient Safety, Agency for Healthcare Research and Quality, Rockville, Maryland; 3 Department of Pediatrics, Johns Hopkins University School of Medicine, Baltimore, Maryland

## Abstract

Antibiotic overuse is common in ambulatory care settings, underscoring the importance of outpatient antibiotic stewardship to ensure safe and effective antibiotic prescription. In response to this need, the Agency for Healthcare Research and Quality (AHRQ) developed the AHRQ Safety Program for Improving Antibiotic Use in Ambulatory Care. The Safety Program successfully assisted 389 outpatient practices across the United States to establish ambulatory antibiotic stewardship. Herein, we have used lessons learned from the AHRQ Safety Program to describe a step-by-step framework to assist practices with establishing antibiotic stewardship in the outpatient setting. Steps include obtaining support from practice leadership; establishing an antibiotic stewardship team; garnering support from practice members; determining how to access antibiotic prescribing data; building communication skills around antibiotic use in the practice; implementing educational content around an infectious syndrome; monitoring antibiotic prescription data; and implementing a sustainability plan.

## The need for outpatient antibiotic stewardship

In the United States, antibiotic-resistant organisms cause 2.8 million infections and 35,000 deaths each year.^1,2^ In the United States, sufficient antibiotic prescriptions are written in the outpatient setting that half of all Americans would receive a prescription in any given year.^3^ Moreover, one-third of antibiotic prescriptions in ambulatory care are considered unnecessary.^3^

Several challenges specific to ambulatory care make judicious antibiotic prescribing difficult. Patient and family preferences have an impact on antibiotic prescribing decisions. Patients may expect antibiotic prescriptions based on prior experiences when presenting with similar symptoms.^4,5^ They may be unaware of the potential harm caused by antibiotics and may not weigh the risks versus benefits associated with antibiotic use.^6^ From the clinician viewpoint, interactions with patients occur during time-constrained office or telemedicine visits, sometimes precluding the ability to collect adequate clinical and diagnostic data prior to the decision to prescribe antibiotics. These brief encounters also pose challenges with balancing competing priorities related to the screening, diagnosis, treatment, and prevention of disease with adequate counseling about antibiotics. For clinicians caring for patients where there may be concerns about their readiness to return to medical care if the clinical status worsens, antibiotics may be prescribed as an attempt to avoid a subsequent complication. In addition, clinician compensation is partially tied to patient satisfaction scores, and many clinicians trying to establish and grow their practices depend on favorable internet and social media reviews from patients.^7^

Understanding the need for antibiotic stewardship in ambulatory settings, the Centers for Disease Control and Prevention (CDC) established the Core Elements of Outpatient Antibiotic Stewardship, which are evidence-based strategies to conduct stewardship in ambulatory settings.^8^ The CDC Core Elements include (1) a commitment to antibiotic stewardship, (2) action for policy and practice to promote appropriate antibiotic prescribing, (3) tracking and reporting to assess progress improving antibiotic prescribing, and (4) education on appropriate antibiotic use for clinicians and patients, with access to colleagues and consultants with additional expertise for specialty care as needed. The Joint Commission developed antibiotic stewardship requirements for accredited ambulatory practices in 2020 that closely resemble the CDC Core Elements. These include (1) identifying an antibiotic stewardship leader; (2) establishing an annual antibiotic stewardship goal; (3) implementing evidence-based practice guidelines related to the antibiotic stewardship goal; (4) providing clinical staff with educational resources related to the antibiotic stewardship goal; and (5) collecting, analyzing, and reporting data related to the antibiotic stewardship goal. Real-world experience on how to successfully implement antibiotic stewardship in ambulatory care consistent with the criteria outlined by the CDC and The Joint Commission remains limited. In response to this need, the Agency for Healthcare Research and Quality (AHRQ) developed the AHRQ Safety Program for Improving Antibiotic Use in Ambulatory Care (ie, the AHRQ Safety Program).

## The AHRQ Safety Program for Improving Antibiotic Use

**Fig. 1.**
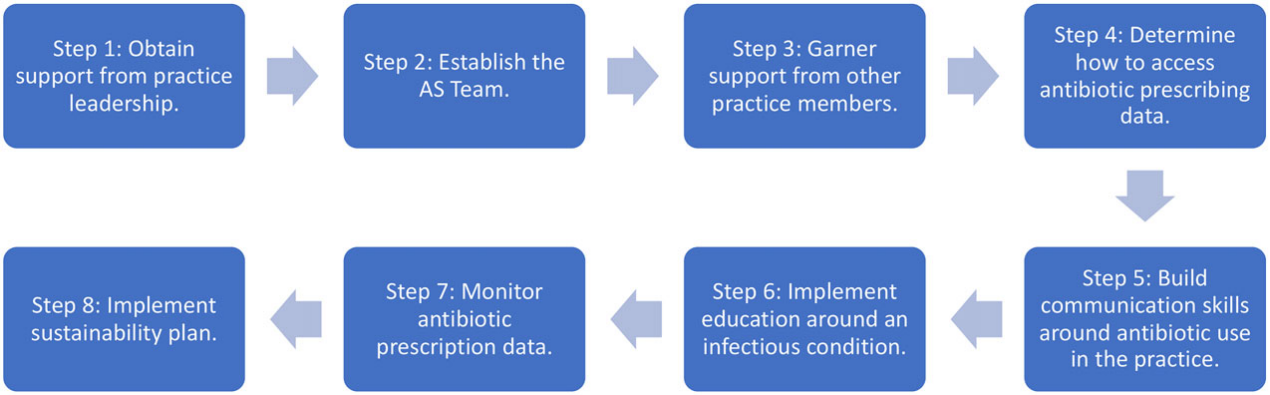
Steps in implementing antimicrobial stewardship programs in ambulatory care.

The overarching goals of the AHRQ Safety Program were (1) to assist ambulatory practices with implementing antibiotic stewardship, (2) to provide frontline clinicians with tools to incorporate antibiotic stewardship principles into routine antibiotic decision making through improving teamwork and communication among practices and between healthcare providers and patients, (3) to provide education on best practices for the diagnosis and treatment of common ambulatory infectious conditions, and (4) to establish the science of safety as an integral component of antibiotic prescribing. In total, 389 outpatient practices including both children and adults from across the United States completed the 1-year AHRQ Safety Program: 162 primary care practices, 160 urgent care practices, 49 federally supported practices, and 18 other practice types.^9^ The AHRQ Safety Program was associated with an overall 50% reduction in antibiotic prescribing and a 36% decrease in antibiotic prescribing for acute respiratory tract infections across participating sites. The content of the Safety Program will soon be publicly available to assist outpatient practices with implementing antibiotic stewardship. The diversity of participating practice types, regional diversity, and types of clinicians observed across practices has provided insight into a framework for implementing antibiotic stewardship in the ambulatory setting (Fig. 1).

## Step 1: Obtain support from practice leadership

Support from senior leadership is an essential first step in implementing antibiotic stewardship in any setting but especially ambulatory care. Senior leadership may include practice owners (including across several practice sites), medical directors, or the most senior clinician in the group, depending on the practice organizational structure. Senior leadership will likely be able to assist with identifying financial or other resources necessary to develop, implement, and sustain antibiotic stewardship interventions. This step may include some protected time for antibiotic stewardship leaders to focus on improving antibiotic prescribing across the practice. Support from senior leadership enables prioritizing information technology (IT) support for electronic data extraction for antibiotic prescriptions, *International Classification of Disease, Tenth Revision* (ICD-10) codes, and other necessary data for the antibiotic stewardship team to measure success over time. Senior leadership can set expectations for other clinic faculty and staff that safe antibiotic prescribing is a priority of the practice. Additionally, obtaining support from senior leadership may be necessary to decouple patient satisfaction scores associated with visits for antibiotic-inappropriate infectious conditions (eg, upper respiratory tract infection, acute bronchitis, influenza without pneumonia, COVID-19) from bonuses and other rewards.

## Step 2: Establish the antibiotic stewardship team

After obtaining support from senior leadership, the antibiotic stewardship team leaders should be identified. One antibiotic stewardship team leader should be a prescribing clinician, such as a physician or an advanced practitioner. Necessary skill sets for clinician antibiotic stewardship leaders include an interest and knowledge in the diagnosis and treatment of common outpatient infections, ability to garner respect from colleagues, ability to be diplomatic and rally members of the practice around antibiotic stewardship goals, ability to communicate clearly and concisely, comfort in the role of educating other practice members, and ability and interest in reviewing data and identifying new approaches to improve antibiotic use.

The other antibiotic stewardship team leader should be an administrative leader who ensures that antibiotic stewardship activities occur in a timely fashion. The administrative leader should have organizational skills that allow for extraction of the necessary electronic data to measure progress of the practice (or the ability to identify someone who can assist with electronic data extraction), ensuring that progress with antibiotic stewardship activities is discussed routinely at practice-wide meetings and that meeting minutes are distributed. Depending on the structure of the practice, the administrative leader may be an office manager, a nurse, a pharmacist, or another practice member.

## Step 3: Garner support from other practice members

To improve antibiotic stewardship in ambulatory care, all members of the practice must prioritize safe antibiotic prescribing. After obtaining support from senior leadership and developing the antibiotic stewardship team, it is important to make the case for antibiotic stewardship to the rest of the practice because antibiotic stewardship activities are more likely to succeed if everyone adheres to them. Understanding of antibiotic stewardship principles should not be limited to practice clinicians but should also include other staff members such as nurses, medical assistants, front-desk staff, and pharmacists. All of these staff members may interact with patients presenting with potentially infectious symptoms and may inadvertently set expectations with patients regarding the likelihood of receiving an antibiotic prescription.

When making the case for antibiotic stewardship to practice members, several points are important to convey. First, emphasize that antibiotic stewardship is a patient safety issue. Remind members of your practice that 19% of all emergency department visits for drug-related adverse events are due to antibiotics.^10^ Although this tradeoff may be acceptable for antibiotics that are necessary, this is not acceptable for antibiotics that are not necessary. Second, remind members of the practice that important downstream consequences of unnecessary antibiotic use may not be immediately apparent, such as increasing antibiotic resistance. Reductions in the diversity of the microbiome and the killing of healthy bacteria increases the likelihood of antibiotic-resistant bacteria that can cause future infections that become increasingly challenging to treat. Third, some of the challenging and dissatisfying conversations with patients who really prefer antibiotics can be avoided if the practice reaches consensus on indications for antibiotic use. By standardizing practices and developing communication tools (including specific phrases to encourage patients to seek appropriate, non-antibiotic based symptomatic treatments), patients will be less likely to seek antibiotics for viral infections from any members of the practice.^6,11–13^ It is important to recognize that the antibiotic prescribing approach of one clinician can impact that of future clinicians. In one study of urgent care practices, receipt of antibiotics for an acute respiratory infection increased the likelihood that both the patient and their spouse would receive an antibiotic for another acute respiratory infection over the subsequent year.^5^

One approach that may help engage practice members in appropriate antibiotic prescribing is an antibiotic commitment poster. Commitment posters require the clinician (by signing or adding their picture to the poster) to make a commitment to prescribe antibiotics judiciously. In this way, the poster not only informs patients and families but also reminds clinicians that they should only prescribe antibiotics for indicated reasons.^14^

## Step 4: Determine how to access antibiotic prescribing data

**Fig. 2.**
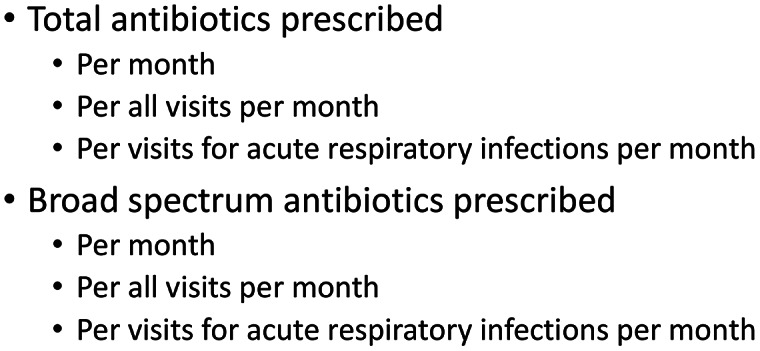
Types of antibiotic prescribing data to collect.

Access to data is an essential part of antibiotic stewardship in ambulatory care, both to monitor progress and to identify new and continued targets in need of further intervention.^8^ Necessary data include antibiotic prescription data (numerator) and the number of clinic visits (Fig. 2, denominator). Additionally, obtaining ICD-10 codes to differentiate conditions for which antibiotic use is expected from those for which antibiotic use is not indicated should be considered. Antibiotic prescription data should be generated monthly (preferably) or quarterly depending on the volume of patients seen at the practice.

Obtaining access to these data may seem challenging; however, electronic health records (EHRs) can streamline the process of electronic data extraction. Practices need access to an IT expert to assist with extracting and tabulating data. An IT expert may be able to develop automated reports for the antibiotic stewardship team to access on an “as needed” basis. Senior leadership may be able to assist with financial resources and with identifying an IT expert. Another option is to contact the EHR vendor to see if they have resources already available to assist with data extraction.

## Step 5: Build communication skills around antibiotic use in the practice

Communication (1) between practice members and patients and families and (2) among practice members is essential in implementing antibiotic stewardship. First, communication skills are important in explaining antibiotic decision making to patients and families. Providing specific statements about why inappropriate antibiotic use can cause harm may help patients and families understand the risks and benefits the clinician is considering in determining whether antibiotics are necessary.^6^ The antibiotic stewardship team can lead the practice in communication skills training that will reduce some of the pressure around unnecessary antibiotic prescribing during routine clinic meetings.

Communication skills training that can reduce unnecessary antibiotic prescribing include the following: (1) encourage use of language that ensures the patient feels heard and that the provider acknowledges the patient is suffering (eg, “I am sorry you are feeling so bad”), (2) communicate negative findings during the physical exam (eg, “Your lungs sound clear so you don’t have pneumonia”), and (3) state the lack of need of an antibiotic as a positive outcome (eg, “The good news is that you don’t need an antibiotic to treat this infection”).^11–13,15^ The antibiotic stewardship team should consider providing clinicians with sample responses to common patient questions about antibiotics.^4,11^

Second, communication among practice members about antibiotic prescribing is essential. Members of the practice must agree on how to manage common infectious syndromes. The antibiotic stewardship team should schedule time to discuss antibiotic stewardship on a regular basis. Finding a time to meet (with full or overbooked clinician schedules) may be difficult, but it is essential in building antibiotic stewardship in ambulatory care. Information provided asynchronously does not replace intrapractice communication about approaches to managing common conditions. Reserving a few moments in a previously scheduled, regular practice meeting (eg, a monthly or quarterly meeting) for which patient visits are already blocked to discuss antibiotic stewardship may be preferred. Practice discussions will facilitate communication and ensure that antibiotic stewardship changes progress.

## Step 6: Implement education around an infectious condition

The antibiotic stewardship team should consider focusing their educational efforts around specific clinical syndromes. In the AHRQ Safety Program, infectious conditions were stratified by those for which antibiotics are always indicated, sometimes indicated, and never indicated. The antibiotic stewardship team may first elect to focus on conditions for which antibiotics are never indicated, such as upper respiratory tract infections, acute bronchitis, influenza without pneumonia, respiratory syncytial virus infections, or COVID-19, because the appropriate antibiotic prescribing rate for these conditions is close to zero. Plan to take several months to focus on education and implementing interventions to improve appropriateness of antibiotic prescribing for the first syndrome selected for focus by the practice, prior to addressing a second high-impact syndrome. As described in step 5, reserve time in a previously scheduled, regular practice meeting to educate clinicians on best practices for the management of the infectious syndrome.

**Fig. 3.**
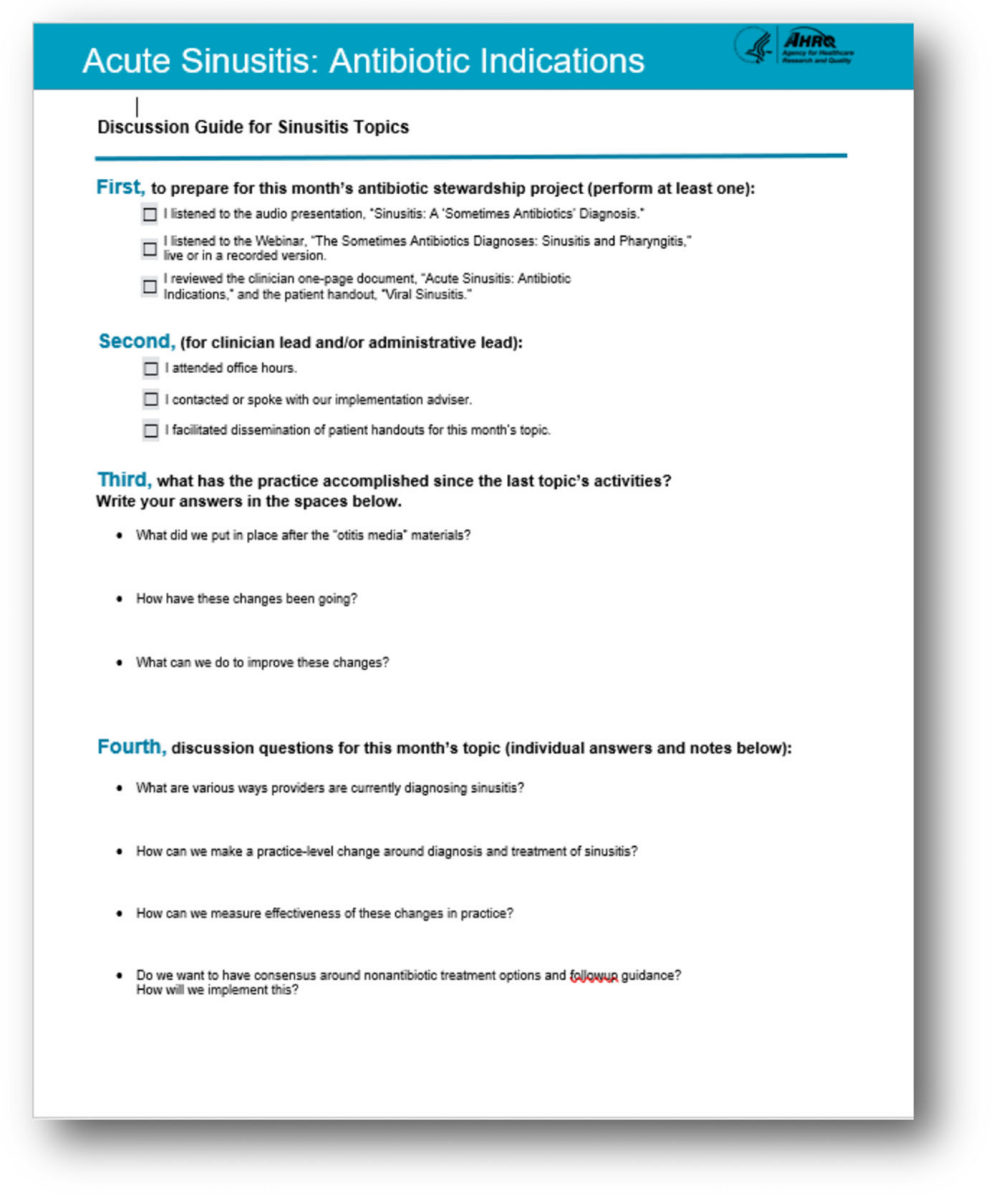
Sample discussion guide.

After selecting the first syndrome to focus on, the clinician antibiotic stewardship leader (as well as a pharmacist if available) should review available evidence-based data on the diagnosis and management of the syndrome. The Safety Program tool kit synthesizes evidence on clinical syndromes (eg, community-acquired pneumonia, skin and soft-tissue infections) through a variety of content, including slide presentations with accompanying facilitator guides that the antibiotic stewardship team can review with the practice, audio presentations, which members of the practice can listen to on their own time, and 1-page documents that summarize each syndrome with sample discussion guides (Fig. 3).

For each clinical syndrome, the safety program content is organized using the “Four Moments of Antibiotic Decision Making” framework. This framework was originally developed in the acute-care setting^16^ and has since been adapted to the ambulatory setting. The Four Moments have been outlined as follows: First, does my patient have an infection that requires antibiotics? Second, do I need to order any diagnostic tests? Third, if antibiotics are indicated, what is the narrowest, safest, and shortest regimen I can prescribe? And fourth, does my patient understand what to expect and the follow-up plan?

Next, after educational material on a syndrome has been reviewed either by a group presentation, listening to audio presentations, or reviewing 1-page documents, the antibiotic stewardship clinician should lead a practice-wide discussion on that syndrome during a regularly scheduled staff meeting to determine what steps the practice can take to improve and standardize their approach to diagnosing and managing that infection. This process may be an iterative one across several monthly meetings and may require reviewing antibiotic prescribing data to ensure changes are occurring (step 7). Once the antibiotic stewardship team and practice members believe sufficient progress has been made on a syndrome, it is reasonable to move on to the next syndrome.

## Step 7: Monitor antibiotic prescription data

After a process has been developed for the antibiotic stewardship team to periodically (monthly or at most quarterly) access antibiotic prescription data, they should review it to determine whether improvements in antibiotic prescribing are occurring and to determine whether additional education or further intervention should be considered. It is helpful to review antibiotic prescription data with the rest of the practice either quarterly or monthly if possible^8^ as part of a regularly scheduled practice meeting so other members can observe the progress achieved over time.

Along with evaluating practice-wide antibiotic prescription data, peer comparison can be an especially impactful antibiotic stewardship tool in ambulatory care.^8^ In peer comparison, antibiotic prescribing feedback is delivered to clinicians so that they can compare their antibiotic prescribing practices with those of their colleagues.^17,18^ Consider informing clinicians whether they are “top performers” (ie, in the top 10%–20% of appropriate antibiotic prescribers) or “not top performers” (ie, all other clinicians).^18^ This approach may incentivize clinicians who are just above the mean to continue to improve in addition to low performers.

**Fig. 4.**
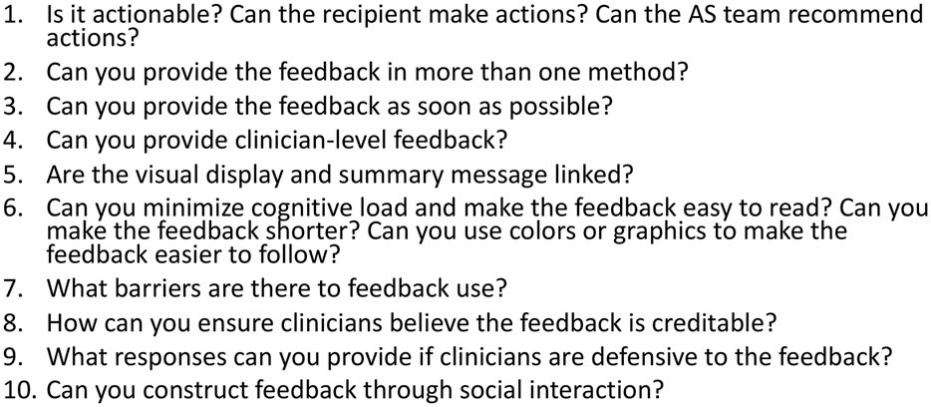
Considerations for audit and feedback.

Providing data to clinicians requires careful thought because many clinicians may express distrust about peer comparison reports.^19^ Therefore, open communication among the practice members at standing meetings about interpreting the reports and how to continue to make improvements in antibiotic prescribing is helpful. Discuss as a practice whether individualized reports should include names of practice members or only the name of the individual for whom the report is intended. Reports should be concise, easy to access, and easy to interpret, or they are unlikely to be reviewed by clinicians.^20^ Use graphics where possible. Recommendations for feedback reports are available (Fig. 4).^21^

## Step 8: Implement sustainability plan

The antibiotic stewardship team should develop a sustainability plan to ensure the longevity of antibiotic stewardship activities. The plan should include the primary goals the antibiotic stewardship team plans to achieve over a designated period (eg, 2 years) as well as an approximate outline of the month to month plans during this time (eg, the order of the syndromes to focus on, the approximate amount of time that will be spent on each syndrome, how frequently to review antibiotic prescription data as a practice). The following aspects should be considered: What processes can become automated (eg, automated electronic data fed into a dashboard), integrated into the electronic health record system (eg, reminders of Centor criteria when considering Group A streptococcal antigen testing),^22^ or delegated from the antibiotic stewardship team to other practice members (eg, peer review of outlier clinicians) to encourage clinician and staff-level ownership of antibiotic stewardship activities? The antibiotic stewardship team should continue to closely oversee all antibiotic stewardship activities. The sustainability plan should also include some general next steps after the 2-year period regarding what the antibiotic stewardship team plans to do to continue to keep the program engaging for both themselves and the rest of the practice. The sustainability plan should be reviewed with senior leadership.

## Additional interventions

Once a practice and antibiotic stewardship team has gone through the above steps, a particular set of initial interventions have been implemented, and a sustainability plan is in place, the practice and the antibiotic stewardship team may consider choosing additional interventions to improve antibiotic prescribing for conditions of interest. For example, practices can implement triage protocols to prevent unnecessary visits for antibiotic-inappropriate conditions through call centers, nurse-managed triage hotlines, or pharmacist consultations.^8^ In these triage protocols, patients calling with symptoms not concerning for severe illness or a condition requiring an antibiotic can be provided symptomatic management over the phone via a nurse. In preventing a visit for a condition for which antibiotics would not be appropriate, patients will be less likely to receive an inappropriate antibiotic. In addition, practices can require written justifications for inappropriate antibiotic prescribing, in which clinicians prescribing an antibiotic for a nonappropriate condition would be required to enter a note in the EHR explaining their decision making.^8,14,18^ Alternatively, practices can implement clinical decision support at the time of documentation or ordering antibiotics.^8^ Other interventions are available, for example, with the CDC Core Elements of Outpatient Antibiotic Stewardship^8^ and with the AHRQ Safety Program Toolkit for Improving Antibiotic Use in Ambulatory Care.

In conclusion, antibiotic stewardship in ambulatory care can be successful, but it requires resources and both a dedicated antibiotic stewardship team and dedicated providers. To assist ambulatory practices in developing and sustaining antibiotic stewardship activities and in following the CDC and The Joint Commission criteria for establishing successful stewardship approaches, we encourage using the tool kit developed through the AHRQ Safety Program for Improving Antibiotic Use in the Ambulatory Care Setting.

## References

[1] CDC. Antibiotic Resistance Threats in the United States, 2019. Atlanta, GA: US Department of Health and Human Services; 2019.

[2] Antibiotic and antimicrobial resistance. Centers for Disease Control and Prevention website. https://www.cdc.gov/drugresistance/pdf/threats-report/2019-ar-threats-report-508.pdf. Published 2019. Accessed June 28, 2022.

[3] Suda KJ , Hicks LA , Roberts RM , Hunkler RJ , Matusiak LM , Schumock GT. Antibiotic expenditures by medication, class, and healthcare setting in the United States, 2010–2015. Clin Infect Dis 2018;66:185–190. 29020276 10.1093/cid/cix773 PMC9454312

[4] Szymczak JE , Keller SC , Linder JA. “I never get better without an antibiotic”: antibiotic appeals and how to respond. Mayo Clin Proc 2021;96:543–546. 33673907 10.1016/j.mayocp.2020.09.031

[5] Shi Z , Barnett ML , Jena AB , Ray KN , Fox KP , Mehrotra A. Association of a clinician’s antibiotic prescribing rate with patients’ future likelihood of seeking care and receipt of antibiotics. Clin Infect Dis 2021;73:e1672–e1679. 32777032 10.1093/cid/ciaa1173 PMC8492129

[6] Miller BJ , Carson KA , Keller S. Educating patients on unnecessary antibiotics: personalizing potential harm aids patient understanding. J Am Board Fam Med 2020;33:969–977. 33219075 10.3122/jabfm.2020.06.200210 PMC7791407

[7] Kohut MR , Keller SC , Linder JA , et al. The inconvincible patient: how clinicians perceive demand for antibiotics in the outpatient setting. Fam Pract 2020;37:276–282. 31690948 10.1093/fampra/cmz066

[8] Sanchez GV , Fleming-Dutra KE , Roberts RM , Hicks LA. Core elements of outpatient antibiotic stewardship. Morb Mortal Wkly Rep 2016;65:1–12. 10.15585/mmwr.rr6506a1 27832047

[9] Keller SC , Caballero TM , Tamma PD , et al. Assessment of changes in visits and antibiotic prescribing during the Agency for Healthcare Research and Quality (AHRQ) Safety Program for Improving Antibiotic Use and the COVID-19 pandemic. *JAMA Netw Open* 2022;5:e2220512. doi: 10.1001/jamanetworkopen.2022.20512. PMC9260475 35793084

[10] Shehab N , Patel PR , Srinivasan A , Budnitz DS. Emergency department visits for antibiotic-associated adverse events. Clin Infect Dis 2008;47:735–743. 18694344 10.1086/591126

[11] Fleming-Dutra KE , Mangione-Smith R , Hicks LA. How to prescribe fewer unnecessary antibiotics: talking points that work with patients and their families. Am Fam Physician 2016;94:200–202. 27479620 PMC6338216

[12] Heritage J , Elliott MN , Stivers T , Richardson A , Mangione-Smith R. Reducing inappropriate antibiotics prescribing: the role of online commentary on physical examination findings. Patient Educ Couns 2010;81:119–125. 20223616 10.1016/j.pec.2009.12.005

[13] Mangione-Smith R , Zhou C , Robinson JD , Taylor JA , Elliott MN , Heritage J. Communication practices and antibiotic use for acute respiratory tract infections in children. Ann Fam Med 2015;13:221–227. 25964399 10.1370/afm.1785 PMC4427416

[14] Meeker D , Knight TK , Friedberg MW , et al. Nudging guideline-concordant antibiotic prescribing: a randomized clinical trial. JAMA Intern Med 2014;174:425–431. 24474434 10.1001/jamainternmed.2013.14191 PMC4648560

[15] Mangione-Smith R , Stivers T , Elliott M , McDonald L , Heritage J. Online commentary during the physical examination: a communication tool for avoiding inappropriate antibiotic prescribing? Soc Sci Med 2003;56:313–320. 12473316 10.1016/s0277-9536(02)00029-1

[16] Tamma PD , Miller MA , Cosgrove SE. Rethinking how antibiotics are prescribed: incorporating the 4 moments of antibiotic decision making into clinical practice. JAMA 2019;321:139–140. 30589917 10.1001/jama.2018.19509

[17] May L, Yadav K, Gaona SD, et al. MITIGATE antimicrobial stewardship toolkit : a guide for practical implementation in adult and pediatric emergency department and urgent care settings. Centers for Disease Control and Prevention. https://stacks.cdc.gov/view/cdc/80653. Published 2018. Accessed June 28, 2022.

[18] Meeker D , Linder JA , Fox CR , et al. Effect of behavioral interventions on inappropriate antibiotic prescribing among primary care practices: a randomized clinical trial. JAMA 2016;315:562–570. 26864410 10.1001/jama.2016.0275 PMC6689234

[19] Szymczak JE , Feemster KA , Zaoutis TE , Gerber JS. Pediatrician perceptions of an outpatient antimicrobial stewardship intervention. Infect Control Hosp Epidemiol 2014;35 suppl 3:S69–S78. 10.1086/677826 25222901

[20] Hemkens LG , Saccilotto R , Reyes SL , et al. Personalized prescription feedback using routinely collected data to reduce antibiotic use in primary care: a randomized clinical trial. JAMA Intern Med 2017;177:176–183. 28027333 10.1001/jamainternmed.2016.8040

[21] Foy R , Skrypak M , Alderson S , et al. Revitalising audit and feedback to improve patient care. BMJ 2020;368:m213. 32107249 10.1136/bmj.m213 PMC7190377

[22] Aalbers J , O’Brien KK , Chan WS , et al. Predicting streptococcal pharyngitis in adults in primary care: a systematic review of the diagnostic accuracy of symptoms and signs and validation of the Centor score. BMC Med 2011;9:67. 21631919 10.1186/1741-7015-9-67 PMC3127779

